# Effect of Xenon Ion Irradiation on the Properties of Austenitic Steel AISI 316

**DOI:** 10.3390/ma17205094

**Published:** 2024-10-18

**Authors:** Piotr Budzyński, Mariusz Kamiński, Zbigniew Surowiec, Marek Wiertel

**Affiliations:** 1 Faculty of Mechanical Engineering, Lublin University of Technology, Nadbystrzycka 36, 20-618 Lublin, Poland; p.budzynski@pollub.pl; 2 Department of Physics, Institute of Physics, Maria Curie-Sklodowska University, Pl. Marii Curie-Skłodowskiej 1, 20-031 Lublin, Poland; zbigniew.surowiec@mail.umcs.pl (Z.S.); marek.wiertel@mail.umcs.pl (M.W.)

**Keywords:** irradiation, crystal lattice, coefficient of friction, wear, fluctuating changes

## Abstract

This study investigated changes in the crystal lattice, tribological properties and friction mechanism of AISI 316 steel irradiated with swift 160 MeV xenon ions. The irradiation process caused the increased roughness of the steel surface and the swelling of the material. The thickness of the irradiated layer increased by about 13 nm. Following irradiation with the fluences 2.5 × 10^14^ and 3.2 × 10^14^ (Xe^24+^/cm^2^), martensite formed in the surface layer. Fluctuating changes were also observed with respect to the coefficient of friction and the degree of wear of the AISI 316 steel samples. Irradiation also increased the microhardness of the steel.

## 1. Introduction

AISI 316 austenitic steel is widely used as a structural material in the food and chemical industries. It is also used for the construction of nuclear reactors and devices operating in high-radiation areas [1,2,3,4,5,6]. The interaction of low-energy ions of elements such as nitrogen, carbon or iron, for example, very often has a positive effect on the mechanical properties of metal alloys by increasing their resistance to corrosion [1,2], microhardness [3], cavitation erosion [4] or tribological wear [5,6,7]. For this reason, ion implantation is used in many industrial solutions as a method to increase the reliability of machines and equipment [8,9]. However, the interaction of highly-energetic particles leads to significant changes in material structure [10,11,12]. Therefore, research into the effects of high-energy particles on the structure and mechanical properties of AISI 316 steel becomes necessary.

The irradiation of AISI 316 with 2 MeV protons at 360 °C causes radiation-induced segregation of its components and radiation hardening [13]. A study [14] investigated the effect of irradiating austenitic steel at temperatures of 200, 300 and 400 (°C) with 8 MeV iron ions on its microstructure and hardness. The results showed a segregation of steel components at grain boundaries and an increase in the evolution of radiation defects with an increase in the irradiation temperature. It was also found that the nanohardness of the steel decreased with an increase in the irradiation temperature. Irradiation with 160 keV iron ions causes an increase in the grain (crystallite) size of AISI 316 [15]. Swelling is widely observed in austenitic steels irradiated with high doses at high temperatures [16]. A study [17] investigated the crystal lattice and defects formed in cold-worked austenitic stainless steel (CW 316 SS) irradiated with 7 MeV Xe^26+^ ions. Shifts in XRD diffraction peaks were observed, indicating an increase in the lattice constant. A study [18] investigated the radiation defects formed in austenitic steel AISI 316 as a result of irradiation at 360 °C with 2 MeV protons to doses up to 6.0 displacements per atom (dpa). The defects induced by proton irradiation included Frank loops, network dislocations, voids and nanotwins. Both the size and the density of the defects increased with the dose.

An analysis of the current state of research indicates that the effects of high-energy ions on the properties of AISI 316 steel are not yet fully understood. In this study, we focused on investigating the impact of xenon ion irradiation on the crystal lattice parameters, tribological properties, microhardness and swelling of AISI 316 grade steel. Our research specifically examined the global effects of irradiation-induced defects on these properties rather than the detailed characterization of the defects themselves.

Furthermore, the influence of varying irradiation doses on the friction and wear mechanisms was explored. The relationship between changes in the crystal lattice structure and tribological properties is well established. For instance, a correlation between the friction coefficient of a Ni monocrystal and its orientation relative to the countersample was reported in [19]. Similarly, for 100Cr6 steel [20] and titanium [21] samples irradiated with 160 MeV xenon ions, fluctuations in the friction coefficient and wear degree were observed to occur in tandem with changes in lattice parameters.

## 2. Materials and Methods

AISI 316 steel, whose chemical composition is given in Table 1, was prepared by Goodfellow Cambridge Ltd. (Huntingdon, UK). Several sets of samples were cut out from AISI 316 and polished. Each set of samples was polished mechanically under the same conditions using a MECATECH 250 SPI polishing machine (Presi, Eybens, France). The surface of the specimens was ground using abrasive papers with gradations from 180 to 600 µm and then polished using 9 µm and 0.3 µm diamond slurries. A mirror-like surface quality was obtained. Some of the samples were annealed in order to remove defects formed during the manufacture of the steel and the preparation of the samples. The chemical composition of this steel grade and that of the countersample (100Cr6) are given in Table 1.

Four sets of samples were prepared from annealed and unannealed steel. The samples were irradiated with 160 MeV xenon ions in swift heavy ion accelerator IC-100. To ensure uniform irradiation of the samples, the ion beam was swept over their surfaces both horizontally and vertically. The irradiation temperature did not exceed 60 °C, and the pressure in the chamber was below 1.1 × 10^−9^ Pa. The unannealed samples were irradiated with the following fluences: 1 × 10^14^, 2.5 × 10^14^ and 3.2 × 10^14^. The annealing samples were irradiated with the fluences 5 × 10^14^ Xe^24+^/cm^2^. Images of the steel surface were captured by atomic force microscopy (AFM) using Nanosurf Easyscan 2 (Nanosurf AG, Liestal, Switzerland). The samples were measured in air. The microscope was installed on active anti-vibration table TS-150. The atomic force microscope cantilevers were calibrated using the Sader method [22]. AFM images of the irradiated layer were processed using WSXM software (WSxM 5.0 Develop 10.3) [23] while the scanning electron microscope (SEM) utilized for this study was the TESCAN Vega 3LMU (Tescan Group, a.s., Brno–Kohoutovice, Czech Republic) equipped with the AZtecEnergy advanced EDX microanalyzer from Oxford Instruments (High Wycombe, UK). The EDX microanalyzer was calibrated using a MAC Universal 55 Reference Standard for X-ray microanalysis. This calibration allowed for element detectability at a level of 0.1% by weight, with measurement uncertainty ranging from 0.01% to 0.09% by weight. For SEM imaging, a backscattered electron detector (BSE) was used.

The coefficient of friction and the degree of wear were measured during tribological tests conducted under technically dry friction conditions with the Anton Paar NTR^2^ tribometer (CFM Instrument) (Anton Paar GmbH, Graz, Austria). The tests were conducted in compliance with the ASTM G99-17 Standard Test Method for Wear Testing with a Pin-on-Disk Apparatus [24]. The countersample was a ball made of 100Cr6 bearing steel (Table 1) with a diameter of 1 mm. The load applied in the tests was 300 mN. The speed of the countersample relative to that of the sample was 5.6 to 6.9 (cm/sec). Wear products were not removed from the friction zone. The degree of wear was measured based on the cross-sectional area of the track made by the countersample. The wear track was measured with the Intra Form Talysurf profilometer (Taylor Hobson Ltd., Leicester, UK) and then processed using Talymap Lite V7 software (ver. 7.4.9431, 2020/10/27). Using die fitted to the irregular shape of the profilograms, the degree of wear was calculated from 15 measurements (profilograms) obtained for each sample at different locations on the track circumference. Microhardness was measured in accordance with ASTM E384-22 [25], with a microhardness meter FM-800 from Future-Tech (Kawasaki, Japan) using the Vickers method with loads of 0.25 N, 1.96 N and 2.94 N. For the Vickers hardness test, the indenter has pyramidal sides with a square base shape with apex angle of θ equal to 136°. In all tests, the loading time was fixed at 10 s. Fifteen impressions were made for each load on each of the test specimens at different depths from the surface of the specimen. The crystal lattice was examined using the Empyrean diffractometer provided with the PIXcel 1D (Malvern Panalytical Ltd., Malvern, UK) silicon strip detector containing 255 strips and covering a range of 3.5° in 2θ scale. This data acquisition system enables fast measurements with high-quality statistics.

## 3. Results and Discussion

### 3.1. Irradiation of AISI 316 Samples

**Figure 1:**
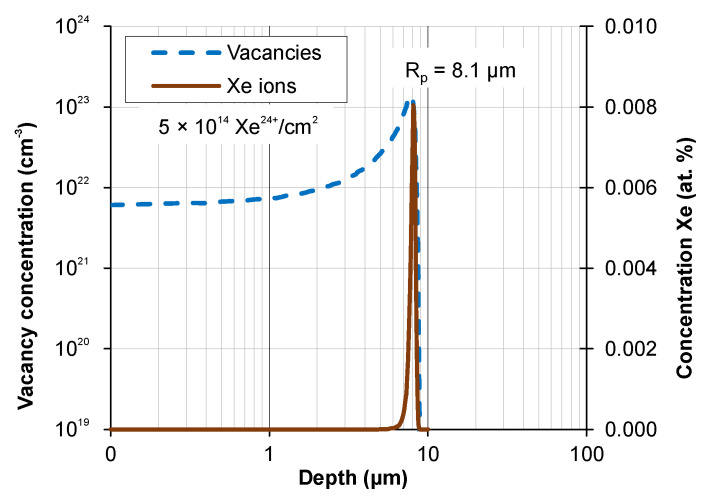
Concentrations of xenon ions and defects induced in AISI 316 by irradiation with a fluence of 5 × 10^14^ Xe^24+^/cm^2^.

**Figure 2:**
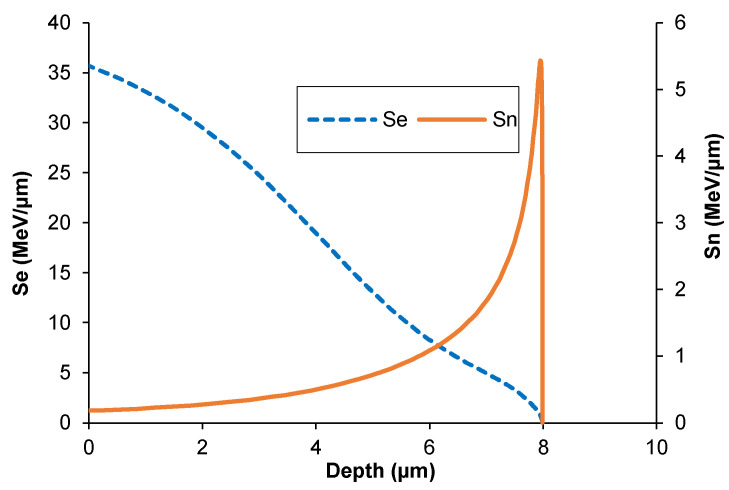
Energy loss of xenon ions during electronic stopping S_e_ and nuclear stopping S_n_ in AISI 316.

Theoretical calculations of the depth distribution of xenon ions and irradiation-induced defects were made using SRIM software (ver. SRIM 2013) [26]—Figure 1. The projected range R_p_ of xenon ions was 8.1 µm. It was assumed that the distribution of radiation defects was reflected by the distribution of vacancies produced during irradiation. The energy required to produce vacancies comes from the energy introduced into the target by xenon ions. Xenon ions were stopped due to the inelastic collisions with the target electrons (S_e_) and the elastic collisions with the nuclei of the target atoms (S_n_). The magnitude of energy transferred into the target during the electronic (S_e_) and nuclear (S_n_) stopping of xenon ions is shown in Figure 2.

**Figure 3:**
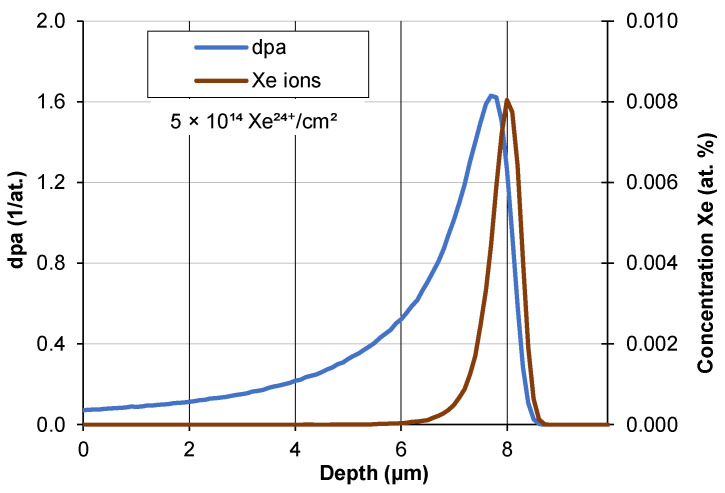
Displacement per atom (dpa) of AISI 316 steel irradiated by xenon ions with a fluence of 5 × 10^14^ Xe^24+^/cm^2^.

The interaction of ions with the target is conveniently described by means of displacement per atom (dpa) units which contain information about the ion fluence and energy as well as the binding energy of the atoms in the target. The dpa number specifies the number of possible displacements of a single atom during bombardment of the target—Figure 3. It does not take into account defect annealing and radiation-accelerated diffusion occurring at room temperature [27,28].

### 3.2. Surface Topography of AISI 316

**Figure 4:**
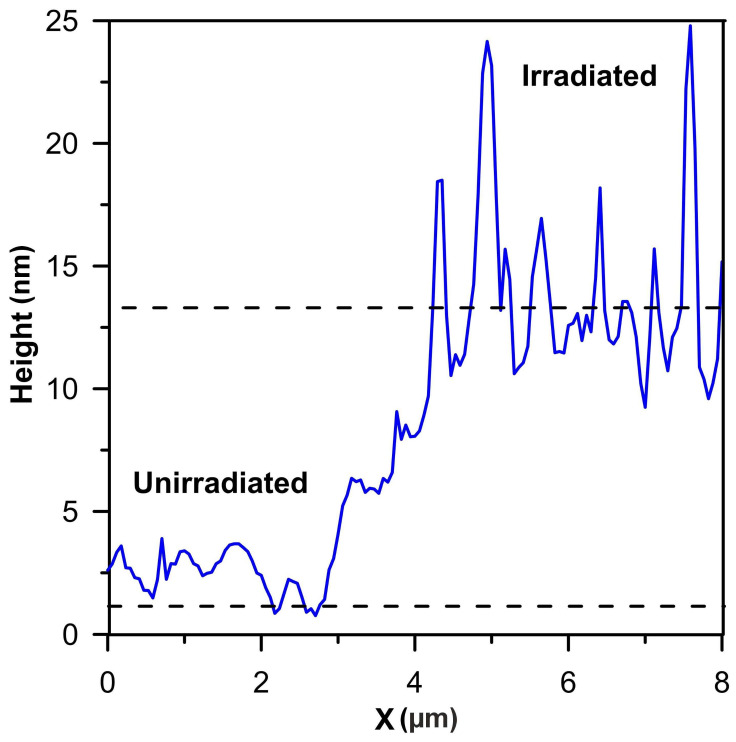
Swelling height of AISI 316 irradiated by xenon ions with a fluence of 5 × 10^14^ Xe^24+^/cm^2^.

The surface of AISI 316 steel before and after irradiation was examined by AFM. One of the profiles is shown in Figure 4. Before irradiation, small irregularities can be seen on the surface, which was obtained after mechanical polishing. After irradiation with a maximum fluence of 5 × 10^14^ Xe^24+^/cm^2^, a number of bands about 10 nm high can be seen. These were formed by the sputtering of part of the surface layer located in between. The sputtering of the target components occurs during elastic collisions of xenon ions with the nuclei of the atoms forming the target. The sputtering rate depends on a number of factors [29,30]: the type of ions, their mass, charge and energy, the relative content of the target components, its crystallographic structure, the orientation of the grains relative to the ion beam, the surface roughness and the radiation-induced surface diffusion.

Irradiation also causes swelling of the sample. The increase in sample thickness is the result of a lattice constant effect, an increase in the number of lattice sites due to diffusion of displaced bulk atoms to the surface and a reduction due to sputtering. After irradiation with xenon ions of 160 MeV energy with a fluence of 5 × 10^14^ Xe^24+^/cm^2^ (1.6 dpa) at T ≤ 60 °C, the swelling height of AISI 316 steel is about 13 nm—Figure 4. In the work [31] AISI 316L steel irradiated at T 350° C with ions of 5 MeV energy with a fluence of 2.6 × 10^15^ ions/cm^2^ (3.7 dpa) had a swelling height of 11 nm. The swelling of AISI 316 steel, despite the high dpa = 3.7, is less than expected due to thermal annealing of some of the defects during irradiation. There is no linear relationship between dpa and the amount of swelling in the sample. Qualitatively, this can be explained within the framework of the multistep damage accumulation model [32,33,34]. In addition, ion-induced annealing may occur [35].

### 3.3. GXRD Measurements

**Figure 5:**
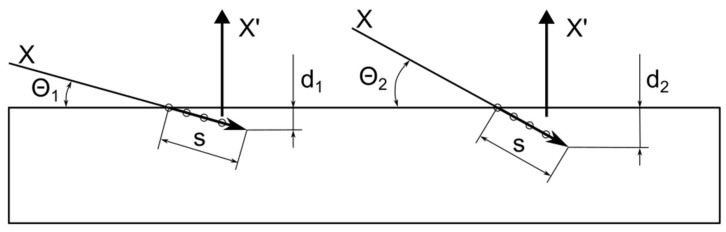
Illustration of the method of testing the crystalline structure of surface layers by GXRD: Q_1_ and Q_2_—angles of incidence of X-rays (X) on the sample surface, s—path length of X-rays in the sample material, which does not depend on the angle of incidence, and d_1_ and d_2_—depth from which scattered X-rays (X′) are emitted.

The grazing incidence X-Ray diffraction (GXRD) method allows the crystalline structure of surface layers of a certain thickness to be studied by varying the angle of incidence (Θ) of X-rays—Figure 5. GXRD measurements were made using a copper lamp with a post-monochromatization X-ray wavelength of 0.1540598 nm.

**Figure 6:**
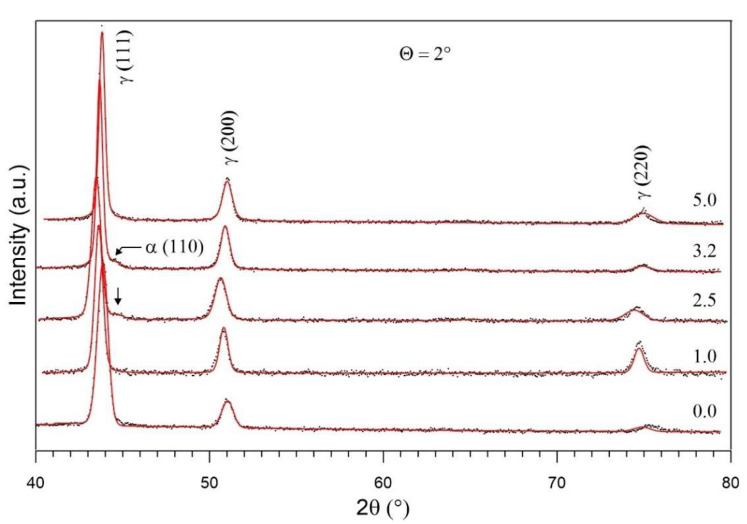
GXRD spectrum measured at Θ = 2°, unirradiated and irradiated by 160 MeV xenon ions with fluences: 1.0, 2.5, 3.2 and 5.0 (×10^14^ Xe^24+^/cm^2^).

Prior to the irradiation of the unannealed sample, a pure austenitic structure (γ) is visible—Figure 6. Characteristic peaks corresponding to the martensitic phase γ (111), γ (200) and γ (220) can be seen, indicating its presence in the irradiated structure. The noticeable changes in peak intensity at different incident angles may suggest some variation in defect distribution and phase transformation as a function of depth. In contrast, the irradiation process of unannealed samples performed with the fluences 2.5 × 10^14^ Xe^24+^/cm^2^ and 3.2 × 10^14^ Xe^24+^/cm^2^, which results in the formation of martensite (α) amounting to about 1.5%. The formation of martensite was observed in a study [36] where the specimens were subjected to solution treatment at 1050 °C for 6 min followed by the water cooling to room temperature. A study [37] also described the transformation of original austenite into martensite in AISI 316L steel subjected to severe shot peening (SSP). There is no martensitic phase in the unheated steel unirradiated and irradiated with a minimum fluence of 1.0 × 10^14^ Xe^24+^/cm^2^. This phase is also absent in the heated steel after irradiation with a maximum fluence of 5.0 × 10^14^ Xe^24+^/cm^2^—Figure 6. This means that the transformation of part of the austenitic phase into a martensitic phase occurs after irradiation with 2.5–3.2 × 10^14^ Xe^24+^/cm^2^ and does not depend on the thermal treatment of the sample before irradiation.

**Figure 7:**
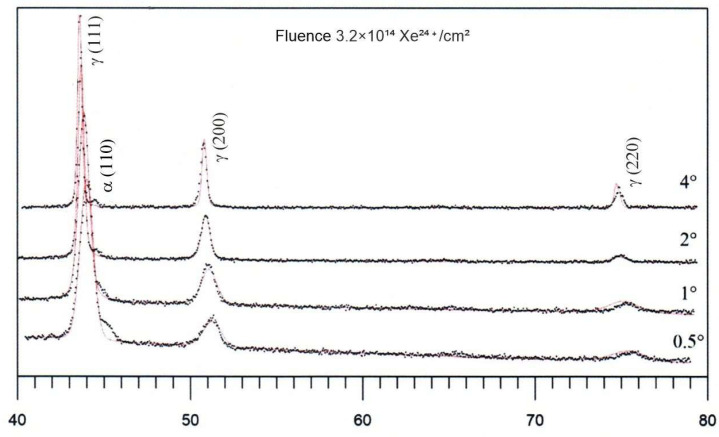
GXRD spectrum measured at Θ = 0.5°, 1°, 2° and 4°, irradiated by 160 MeV xenon ions with fluence 3.2 × 10^14^ Xe^24+^/cm^2^.

After irradiation of the unannealed samples with a fluence of 3.2 × 10¹⁴ Xe²⁴⁺/cm², X-ray diffractograms measured at different incident angles (0.5°, 1°, 2° and 4°) show the presence of a martensitic phase throughout the irradiated layer of AISI 316 steel—Figure 7. Characteristic peaks corresponding to the martensitic phase γ (111), γ (200) and γ (220) can be seen, indicating its presence in the irradiated structure. The noticeable changes in peak intensity at different incident angles may suggest some variation in defect distribution and phase transformation as a function of depth.

Fluctuating changes in the position of the GXRD spectrum peaks of austenite (111), (200) and (220) occur in the same phase with changes of the irradiation fluence—Figure 6. This means that the lattice constant changes with the increase of the irradiation fluence (Table 2). The changes in the lattice constant of layers located at different depths, depending on the incident angle Θ and irradiation fluence, are given in Table 2. The most pronounced changes were observed for the samples irradiated with a fluence of 2.5 × 10^14^ Xe^24+^/cm^2^ for all incident angles. Irradiation with fluences of 1 and 2.5 × 10^14^ (Xe^24+^/cm^2^) led to a greater increase in the lattice constant than irradiation with higher fluences.

A small increase in the lattice constant can be observed after high fluence irradiation. The lack of a linear relationship between irradiation fluence and changes in the lattice constant may be due to the gradual accumulation of defects and their evolution after reaching a characteristic density [32,33,34]. The lack of such a relationship may be partly attributable to partial quenching of defects during irradiation with a higher ion fluence [35]. The subsequent ion portion may partially rebuild radiation damage caused during the previous irradiation step.

The range of X-rays in a given target depends on their energy and the absorption capacity of the target. By changing the incident angle, we can change the depth from which the signal reaches the detector.

### 3.4. Coefficient of Friction and Wear of Steel

**Figure 8:**
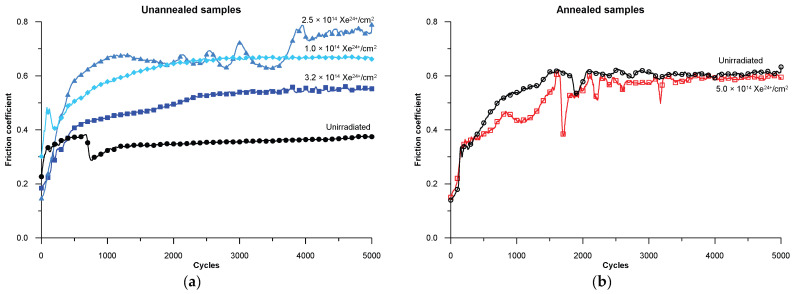
Changes in the coefficient of friction for the AISI 316 samples during tribological testing for (**a**) unannealed samples and (**b**) annealed samples [38].

The results of the coefficient of friction from the tribological tests are shown in Figure 8. The lowest coefficient of friction was obtained for the unannealed and unirradiated steel samples, i.e., those with only defects formed during standard sample preparation—cutting, grinding and polishing. The coefficient of friction for the unannealed and unirradiated samples becomes stable at 0.35 above 2000 measurement cycles. Following irradiation with fluences of 1, 2.5 and 3.2 (×10^14^ Xe^24+^/cm^2^), there is a significant increase in the coefficient of friction value. The changes are fluctuating and depend on the irradiation dose. The largest fluctuations in the coefficient of friction during the tribological test are observed for the steel irradiated with a fluence of 2.5 × 10^14^ Xe^24+^/cm^2^. Irradiation with this fluence value maximally increases the lattice constant in all the tested layers (at different depths)—Table 3. The observed increase in the coefficient of friction for the AISI 316 steel specimens irradiated with high-energy xenon ions is similar to the exposure to the same Xe ion radiation of 100Cr6 bearing steel [20]. The increase in the coefficient of friction of the irradiated samples is often attributed to an increase in stresses in the surface layer and increased surface roughness caused by the impact of large and fast xenon particles.

The annealed samples have a relatively high coefficient of friction, with its value becoming stable at 0.6 above 2000 measurement cycles. Irradiating the samples with a 5 × 10^14^ Xe^24+^/cm^2^ fluence causes high fluctuations in the coefficient of friction during tribological tests. The defects produced during mechanical sample preparation significantly reduce the friction coefficient—Figure 8a, compared to annealed samples—Figure 8b. Radiation defects created after irradiation increase the friction coefficient of the material as compared to the non-irradiated material. The friction coefficient of the annealed samples after irradiation with a fluence of 5 × 10^14^ Xe^24+^/cm^2^ shows large local changes—Figure 8b.

**Figure 9:**
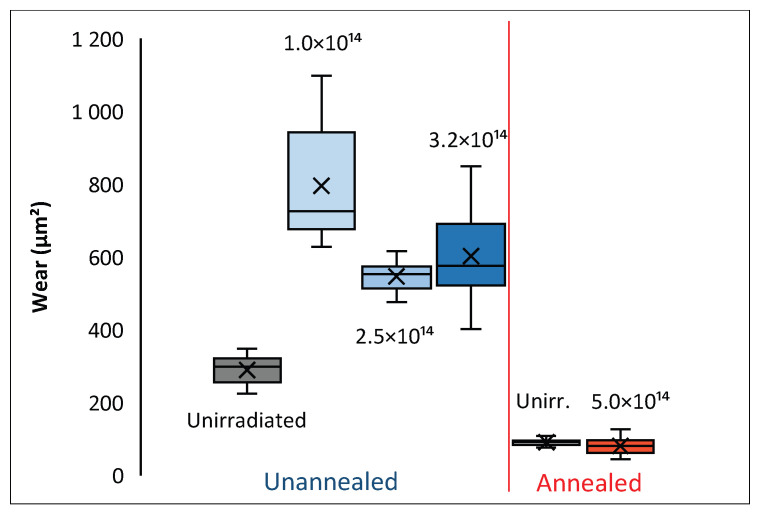
Wear of samples after 5000 measurement cycles of a tribological test.

After a 5000-cycle tribological test, profilometric measurements of the wear track on the sample were conducted, and based on these measurements, the average wear for each sample was determined. The results are presented in Figure 9. The lowest wear is obtained for the unirradiated AISI 316 samples after annealing. The use of irradiation does not change the degree of wear of the annealed samples, just as it did not change the coefficient of friction above 2000 measurement cycles—Figure 8a.

### 3.5. SEM Images of Countersamples

**Figure 10:**
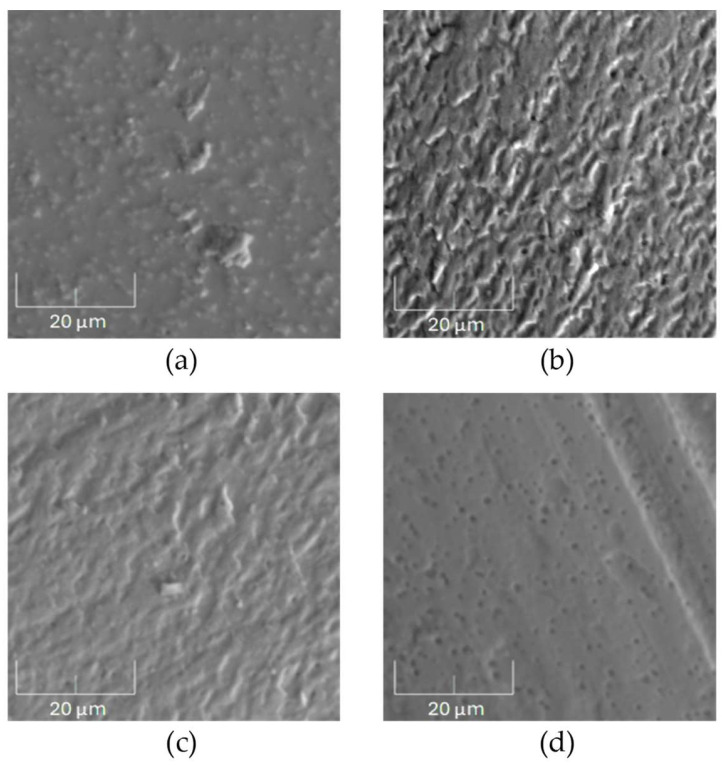
SEM images of the wear track on the surface of the 100Cr6 countersamples after tribological testing of the unannealed AISI 316 steel samples: (**a**) unirradiated, (**b**) irradiated with a fluence of 1 × 10^14^ Xe^24+^/cm^2^, (**c**) 2.5 × 10^14^ Xe^24+^/cm^2^ and (**d**) 3.2 × 10^14^ Xe^24+^/cm^2^. Magnification: 5000×.

SEM images of the wear track on a 100Cr6 steel countersample interacting with the unannealed AISI 316 steel samples during the tribological test are shown in Figure 10. On the countersample surface, the presence of abraded fragments can be observed from both the unirradiated sample (Figure 10a) and the sample irradiated with a 1 × 10^14^ Xe^24+^/cm^2^ fluence (Figure 10b). These abraded fragments remain on the countersample’s surface during the test and are pressed against it, leading to further wear of the sample. The presence of wear products near the wear track may be facilitated by weak magnetic interactions of the abraded fragments with the countersample. A study [39] showed that defects in SiC material fragments could induce magnetic interaction. Irradiation with a higher fluence leads to increased microhardness and abrasive wear of the sample. The SEM images in Figure 10c and partially in Figure 10d shows characteristic stripes on the surface of the countersamples that were abraded by the samples with increased microhardness, which is confirmed by the microhardness measurement data of the samples described later in this paper.

**Figure 11:**
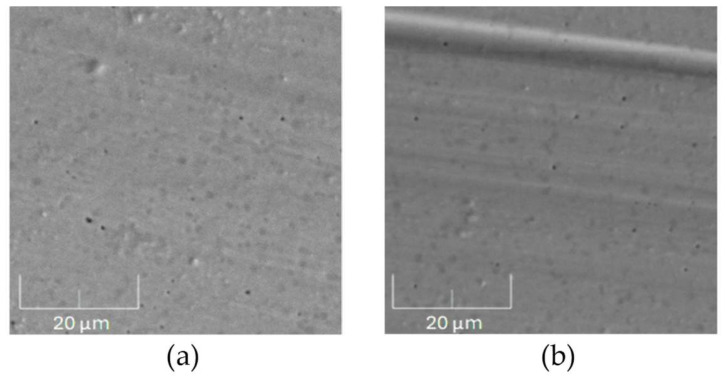
SEM images of the wear track on a 100Cr6 countersample after tribological tests conducted on annealed the AISI 316 samples: (**a**) unirradiated and (**b**) irradiated with a fluence of 5 × 10^14^ Xe^24+^/cm^2^. Magnification: 5000×.

Figure 11 shows SEM images of the wear track on the surface of the countersamples after tribological tests conducted on the annealed AISI 316 steel samples. Figure 11a shows small fragments of the annealed unirradiated sample deposited on the surface of the counterexample. Small holes from the chipped counterexample material can also be seen. After irradiation with a 5 × 10^14^ Xe^24+^/cm^2^ fluence, the microhardness of the annealed AISI 316 steel increases, carving characteristic stripes on the surface of the counterexample during the tribology test. This observation is confirmed by the data in Table 3.

### 3.6. Microhardness

**Figure 12:**
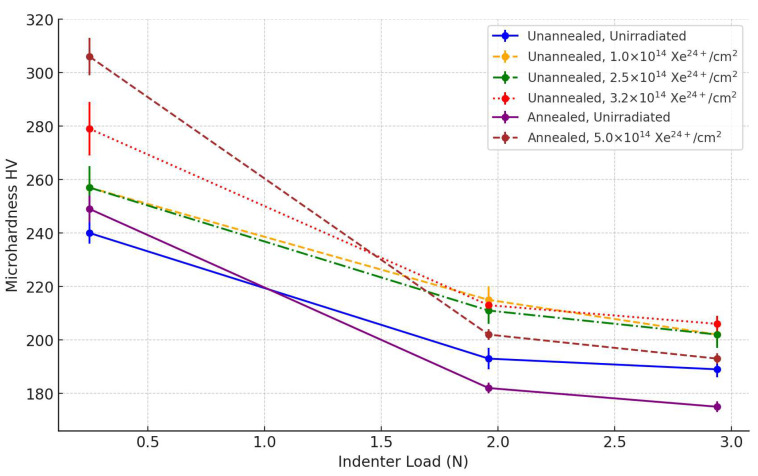
Average microhardness values of tested AISI 316 steel specimens at different indenter loads.

Microhardness tests on AISI 316 steel measured at different loads before and after implantation of Xe^24+^ ions showed clear changes that can be attributed to both direct implantation effects and the changes in the material’s crystalline structure found earlier. These results, illustrated in Table 3 and Figure 12, show different changes depending on the indenter load (0.25 N, 1.96 N and 2.94 N).

Microhardness measurements performed with a load of 0.25 N, where the indentation depth did not exceed 3 µm, showed that the implantation of Xe ions caused a significant increase in microhardness. For the non-annealed samples, the microhardness value increased from 240 HV to 279 HV, with a highest fluence of 3.2 × 10^14^ Xe^24+^/cm^2^. A similar increase was observed in the annealed samples, where the hardness reached 306 HV after implantation at a fluence of 5 × 10^14^ Xe^24+^/cm^2^. This significant change in surface hardness can be directly related to the structural changes found in the GXRD tests. The formation of martensite in this zone, which is a harder phase than the original austenite, contributes to the increased resistance to plastic deformation, leading to an increase in microhardness. In addition, changes in the crystal lattice constant and fluctuations in the austenite peaks (111), (200) and (220) suggest the existence of significant lattice stresses, which can also strengthen the surface layer of the material.

Measurements made with a load of 1.96 N indicate that the imprint depth of the irradiated samples does not exceed 8.5 μm. At this depth, according to SRIM simulation results, the maximum concentration of implanted ions and defects is located. Also, for these measurement parameters, a significant increase in microhardness was observed compared to samples without implantation. In the unannealed samples, the microhardness increased from 193 HV to 213 HV at a fluence of 3.2 × 10^14^ Xe^24+^/cm^2^. In the annealed samples, the value increased from 182 HV to 202 HV after implantation with a fluence of 5 × 10^14^ Xe^24+^/cm^2^.

These changes are consistent with observations of an increase in crystallite size and a reduction in crystal lattice deformation at this depth, as demonstrated by GXRD. The formation of defects in this zone, including vacancies and atomic clustering, contributes to the strengthening of the material by reducing dislocation mobility, which in turn increases microhardness. Furthermore, the observed changes in the crystal lattice constant may affect the local stress concentration, which further contributes to the increase in hardness at this depth. A similar phenomenon of an increase in microhardness at the depth corresponding to the highest ion concentration was found in certain works [40,41]. An increase in the microhardness of AISI 316L steel beyond the range of high-energy protons can be seen in Figure 7 of the paper [13].

At the highest applied indenter load (2.94 N), the indenter was observed to penetrate to a depth of 10.5–10.6 um for unirradiated samples that had been irradiated with Xe ions. The irradiated and previously annealed sample showed a depression of approximately 10.8 um. These are above the original range of Xe ions. Changes in microhardness at a depth of 10 µm are less pronounced, indicating that the effect of irradiation on the deeper layers of the material is limited and may be due to the influence of the surface layer during microhardness measurement. A similar increase in the nanohardness of 2 MeV proton-irradiated AISI 316 steel was also observed in [13] at a depth greater than the implanted ion range calculated with the SRIM package [26].

### 3.7. EDX Measurements

**Figure 13:**
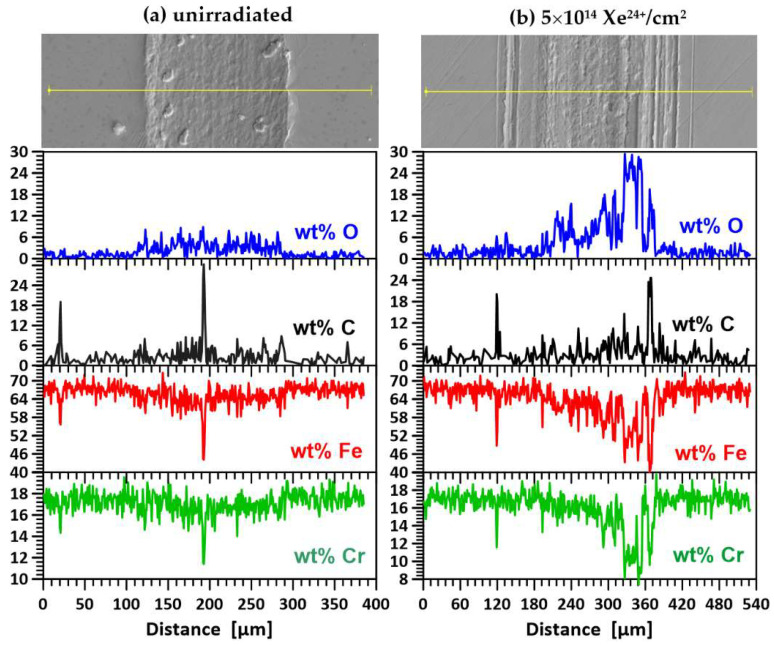
Images of the surface and wear track of the annealed steel AISI 316 samples with a scan line for elemental mapping: (**a**) unirradiated and (**b**) irradiated with a fluence of 5 × 10^14^ Xe^24+^/cm^2^.

SEM images of the wear track on the annealed samples during the tribological test are shown in Figure 13. The line drawn in the figure represents a scanning path along which the contents of oxygen, carbon, iron and chromium were measured. Oxygen and carbon are elements that can significantly influence the friction process, while iron and chromium are the main components of steel. In the annealed samples, reduced abrasion resistance is evident, as shown in Figure 13a. Large sections of the sample material are pulled out, indicating the presence of adhesive and abrasive wear accompanied by oxidative wear. This is consistent with the increased oxygen content detected along the wear track, suggesting the formation of surface oxides that exacerbate material degradation.

The irradiation process significantly alters the wear track morphology, as shown in Figure 13b. Grooves, mounds and small clusters of displaced material are visible, pointing to a more complex wear mechanism. The increased oxidative wear is likely due to enhanced oxygen diffusion along radiation-induced defects, allowing oxygen to penetrate deeper into the sample and promote oxidation. This phenomenon can be attributed to the high fluence of xenon ions, which created diffusion pathways for oxygen through radiation-induced defects. Radiation significantly influences oxygen diffusion in materials, particularly steel, by increasing point defect concentrations and enhancing diffusion pathways [42,43,44]. Irradiation of heated samples alters the friction process. Adhesive wear decreases, and oxidative wear increases. It is interesting to note that the amount of grated material does not change during the tribological test.

**Figure 14:**
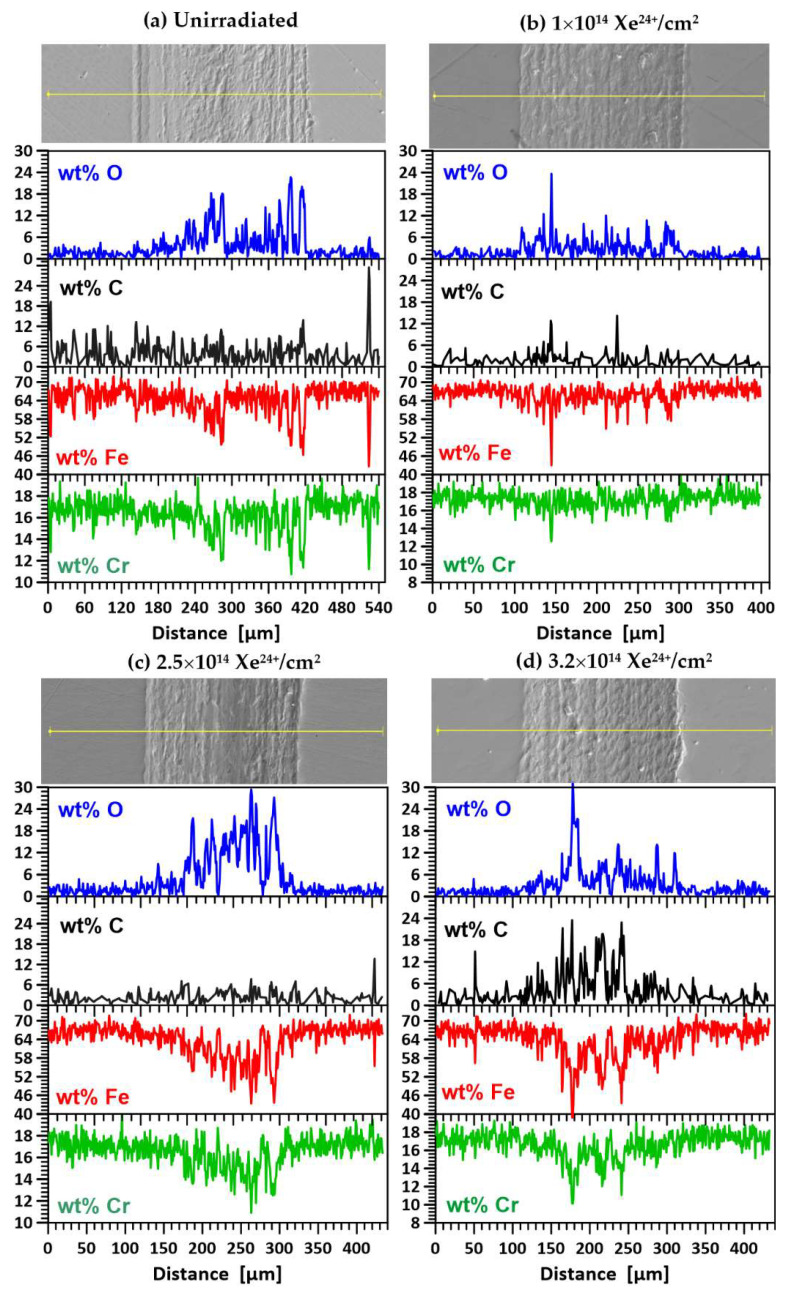
Images of the surface of the unannealed AISI 316 samples together with a scan line for elemental mapping before irradiation (**a**) and after irradiation with fluences: (**b**) 1 × 10^14^ Xe^24+^/cm^2^, (**c**) 2.5 × 10^14^ Xe^24+^/cm^2^ and (**d**) 3.2 × 10^14^ Xe^24+^/cm^2^.

EDX analysis revealed the presence of carbon on the surface of samples—Figure 13 and Figure 14. This originated from residual oil vapour deposited during EDX measurements despite the turbomolecular pumps used to minimize contamination of the sample surfaces. In contrast, the carbon at the bottom of trace—Figure 14d (an unheated sample irradiated with a fluence of 3.2 × 10^14^ Xe^24+^/cm^2^) was transferred from the 100Cr6 pre-sample due to chemical reactions with Fe, Co and Ni to form carbides of these metals. These reactions were favoured by the high temperature, as evidenced by the melting of the surface of the counterexamples during the tribological test. This is particularly evident in Figure 10b,c. The surface of the samples was also remelted—see the SEM microphotograph in Figure 14d. Several depressions can be seen on the surface of the trace, indicating local suturing of the sample and countersample fragments. In addition, the friction coefficient of the unheated sample after irradiation with a flux of 3.2 × 10^14^ Xe^24+^/cm^2^ is the lowest among the irradiated samples and has a rather smooth course—Figure 8a. The presence of carbon can cause a reduction in the friction coefficient. Its implantation has long been used to improve the tribological properties of steels [45].

Figure 14a–d show SEM images of the wear track on the unannealed samples during the tribological test and the results of oxygen (O), carbon (C), iron (Fe) and chromium (Cr) measurements along the wear track. For the unirradiated specimens (Figure 14a) and those irradiated with doses of 1 × 10^14^ Xe^24+^/cm^2^ (Figure 14b) and 2.5 × 10^14^ Xe^24+^/cm^2^ (Figure 14c), the wear mechanism did not change significantly compared to the unirradiated specimen. EDX analysis showed no significant differences in the elemental content of the wear trace. Wear was still of an oxidative, abrasive and adhesive nature, as in the unimplanted sample.

Only implantation with a dose of 3.2 × 10^14^ Xe^24+^/cm^2^ (Figure 14d) caused significant changes in the wear mechanism. SEM microphotography shows an increase in adhesive wear and remelting of the sample surface. Adhesive wear is confirmed by an increase in the carbon content of the wiped trace on the sample surface, which originates from the countersample.

## 4. Conclusions

The irradiation of AISI 316 steel with 160 MeV xenon ions causes an increase in surface roughness, an increase swelling, an increase in the lattice constant value and changes in the mechanical properties of the modified layer. The surface roughness increase is due to different sputtering rates of the steel components. The changes in the surface condition induced by irradiation with a 5 × 10^14^ Xe^24+^/cm^2^ fluence can be seen in the AFM image—Figure 3. Irradiation increases the thickness of the modified layer by 13 nm. These changes result from an increase in the lattice constant and an increase in the number of lattice sites due to diffusion of displaced bulk atoms to the surface and a reduction due to sputtering.

The lattice constant decreases with an increase in the irradiation fluence. A similar effect was reported in a study [11]. The displacement of the γ peak (111) toward smaller angles 2θ in the irradiated CW316SS steel decreases after irradiation with the maximum fluence. The smaller increase in the lattice constant after higher fluence irradiation may be due to the annealing of some radiation defects produced during the nuclear and electronic stopping of xenon ions. During irradiation with a 5 × 10^14^ Xe^24+^/cm^2^ fluence, a single atom of AISI 316 located at a depth of 7.8 μm theoretically can statistically change its lattice site an average of 1.6 times.

The coefficient of friction and the degree of wear of the AISI 316 steel samples depend on a number of factors: the lattice constant value, microhardness and the interaction of the countersample with the sample. The maximum increase in the lattice constant after irradiation with a fluence of 2.5 × 10^14^ Xe^24+^/cm^2^ is correlated with the maximum coefficient of friction and the maximum fluctuations in the coefficient of friction during the tribological test.

Implantation of Xe^24+^ ions with an energy of 160 MeV leads to significant changes in the microhardness of AISI 316 steel, with the greatest changes observed at relatively low loads at depths corresponding to the maximum concentration of implanted ions and structural defects (3 µm and 8 µm). The increase in hardness is closely related to changes in the structure of the crystal lattice, including the formation of martensite, an increase in crystallite size and a reduction in lattice stresses, as demonstrated by GXRD studies. Changes in microhardness at a depth of 10 µm are less pronounced, indicating that the effect of implantation on the deeper layers of the material is limited. They can be caused by the influence of the surface layer when measuring microhardness.

Changes in tribological properties induced by irradiation depend on several factors: surface topography, crystal lattice constant and lattice strain, crystallite size, microhardness and the interaction of the AISI 316 steel components with the countersample material. The combined effect of these factors leads to fluctuations in tribological changes depending on irradiation fluence.

## Figures and Tables

**Table 1:** Chemical composition of steel grades of AISI 316 (sample) and 100Cr6 (countersample).

Element (wt. %)/Steel	C	Cr	Mn	Mo	Si	Ni	Cu	P	S	N	Fe
AISI 316	<0.07	17.5	1.9	2.0	<1	12.5	-	<0.04	<0.02	<0.01	balance
100Cr6	1.1	1.5	0.30	≤0.10	0.25	≤0.40	≤0.30	<0.03	<0.03	-	balance

**Table 2:** Changes in the lattice constant Δa (nm) for different irradiation fluences and incident angles versus unirradiated bulk sample; d—thickness (depth) of tested layer.

Fluence (Xe^24+^/cm^2^)/Δa (nm)	Θ = 0.5°d = 0.097 µm	Θ = 1°d = 0.194 µm	Θ = 2°d = 0.388 µm	Θ = 4°d = 0.776 µm
1.0 × 10^14^	0.0014 (2)	0.0013 (2)	0.0020 (2)	0.0011 (2)
2.5 × 10^14^	0.0012 (2)	0.0016 (2)	0.0025 (2)	0.0016 (2)
3.2 × 10^14^	0.0004 (2)	0.0011 (2)	0.0008 (2)	0.0001 (2)
5.0 × 10^14^	0.0004 (2)	0.0015 (2)	0.0003 (2)	0.0002 (2)

**Table 3:** Changes in the microhardness of the AISI 316 steel samples after irradiation.

Sample	HV 0.025	HV 0.2	HV 0.3
Unannealed, unirradiated	240 ± 4	193 ± 4	189 ± 3
Unannealed, 1.0 × 10^14^ Xe^24+^/cm^2^	257 ± 7	215 ± 5	202 ± 3
Unannealed, 2.5 × 10^14^ Xe^24+^/cm^2^	257 ± 8	211 ± 5	202 ± 5
Unannealed, 3.2 × 10^14^ Xe^24+^/cm^2^	279 ± 10	213 ± 3	206 ± 3
Annealed, unirradiated	249 ± 5	182 ± 2	175 ± 2
Annealed, 5.0 × 10^14^ Xe^24+^/cm^2^	306 ± 7	202 ± 2	193 ± 2

## Data Availability

The data presented in this study are available on request from the corresponding author.
